# Clinical Society of Maryland

**Published:** 1894-03

**Authors:** H. O. Reik

**Affiliations:** Secretary; 525 N. Howard St.; Baltimore


### Society Reports.

### CLINICAL SOCIETY OF MARYLAND.

BALTIMORE, February 2, 1894.

The two hundred and ninetieth meeting was called to order by the President, DR. J. E. MICHAEL.

DR. P. C. WILLIAMS, in his remarks on the use of "Tarnier forceps in occipito-posterior positions," said: In my early days nearly all the ruptured perineums I saw were due either to post-occipito positions or to delivery of the shoulders. Two of the most advantageous points about this instrument are the ball joints at the handle and at the traction rods. As you pull, the handles begin to rise and then to rotate. When the head has reached the floor of the pelvis it is advisable to remove the forceps, detach the rods and again apply the blades in their usual position; otherwise you are apt to injure the soft parts. The first time I used Tarnier's forceps was in the delivery of a child with very large head from a rather small woman. As soon as I began traction rotation took place and I had an easy delivery.

The larger the head, the more certain is it that rotation will occur during traction. I consider them the ideal forceps for all high positions of the head. They can be used *in utero* without any trouble.

DR. WILMER BRINTON read a paper on "Recent experiences with axis traction forceps."

*Case* 1.—Louisa Jackson, colored, was admitted to the Maryland Lying-in Hospital June 8th, 1893. She was pregnant with her first child; age, nineteen years. She made the statement that the "first day of her last sickness" was October 5th, 1892. Within two weeks after she entered the hospital, in the presence of a class of students, I measured her pelvis, using for this purpose the Schultze pelvimeter and my hand. I found the transverse diameter normal, but that there existed a marked contraction of the anteroposterior diameter of the superior strait, being three and one-half inches. At this time and in the presence of students, I stated that from the measurements, I was sure that the woman would have a difficult labor, but that if nature did not do the work, that forceps would deliver her, without the necessity of other operative measures on mother or child. Labor pains began a few minutes past midnight July 20th, 1893. An examination then made by my assistant found the child presenting vertex, with the occiput to the mother's front and left (occipito-læva anterior). From this time on the pains continued with more or less severity. At 3 : 30 P. M., some fifteen hours from the beginning of true labor, the membranes ruptured spontaneously. At 5:30 P. M. a hypodermic injection of one-fourth grain of morphia was given. She then slept "off and on" until eight o'clock, when her pains began again, and were of a severe character. I first saw this woman at midnight, after she had been in labor twenty-four hours. I found her in a good condition, pulse being about 100°, but she complained of feeling thoroughly exhausted. An examination per vaginam, found the cervix about half dilated ; the child's head above the superior strait and movable ; the heart sounds of the child, which were heard to the mother's left side, indicated that the child was in no immediate danger. Under the existing condition, we determined to postpone operative measures until early next morning. Hydrate of chloral, grains fifteen, was now given every twenty minutes until a drachm was taken, and from 3:30 o'clock in the morning until I saw her at 6 o'clock, she had hot vaginal douches every thirty minutes. When my colleague, Dr. Crouch, and myself examined this patient at this hour, we found no material change from the examination made at midnight, with the exception, of the cervix being more dilated and dilatable. The head was still movable, the mother was in quite an exhausted condition, and we determined on operative measures in the interest of both mother and child. What should it be ? Version or high forceps ? With some predilection for version in this class of cases, yet we determined on high forceps. The woman was thoroughly chloroformed and Lusk's modification of Tarnier's forceps was applied. After considerable traction, lasting "off and on" for about forty minutes, we succeeded in delivering her of a living female child. A tear of the perineum was immediately repaired. A brief history of the case as taken from the hospital record book is as follows: First stage of labor twenty-nine hours, second stage fifty minutes, third stage ten minutes, presentation vertex position O. L. A., child female, weight six and one-half pounds, length eighteen inches. The child was nursed by its mother, who left the hospital August 3d, 1893, after an uneventful lying-in period.

*Case* 2.—At two A. M., January 22d, 1894, my associate, Dr. J. F. Crouch, was called to see Mrs. T., a native of Ireland, age thirty-four years. He found her in labor for the second time. She had had true labor pains for two days before the doctor was called; the membranes had ruptured spontaneously forty hours before. From an inquiry made at this visit it was ascertained that her first labor must have been a very difficult one. She had been under the care of a midwife for nearly two days, when finally a doctor was sent for, and after great exertion upon his part she was delivered of her child, which only lived a few minutes. An interval of two years elapsed, when she conceived again, and, as stated, after having been in labor for nearly two days, Dr. Crouch was then sent for. He found the child presenting vertex, head high up and movable, fetal heart heard distinctly to the mother's left. The "os" was found dilated to the "size of a half dollar." The pains continued all day Monday without progress. I first saw this woman at five o'clock in the afternoon, some fourteen hours after Dr. Crouch first saw her, and nearly three days from the time she first went into labor. I found the patient in an exhausted condition, pulse 128 and bad. She was extremely restless, rolling from one side of the bed to the other. A vaginal examination found the parts hot and dry, the cervix dilated to the size of a silver dollar, but rigid and œdematous, the presenting part, in my opinion, being a brow. While examining I pushed up the presenting part, and in the light of subsequent events I believe I caused enough flexion to transpose what was undoubtedly a partial brow into a vertex presentation. While thus engaged in pushing up the presenting part I had no trouble in making out with my fingers a marked deformity; the promontory of the sacrum projected to such an extent that the anteropost diameter of the superior straight was estimated to be less than three and one-half inches. We revisited our patient at eight o'clock P. M., found no progress, pulse more rapid, exhaustion very decided, the cervix more œdematous, the head still movable above the brim of the pelvis. We decided to delay no longer. Chloroform was given to full anesthesia. Neale's Axis Traction forceps were applied, and after considerable traction a medium sized male child was delivered. The child was asphyxiated, but was soon restored to animation by the usual methods. The placenta was delivered by the Crede method fifteen minutes later. The uterus contracted and retracted well, but considerable blood was lost, but the hemorrhage was soon under control by kneading the uterus—hand in uterus and uterine injections of hot water. The patient has had no trouble during her lying-in period, and now on her eleventh day is going about her room. The child still shows evidence of pressure about the cranium, but is prospering and will do well. As we never saw the woman until we were called to see her after being in labor two days, we did not have the opportunity of measuring her pelvis. We hope to do this accurately within a fortnight, and we shall report the results later. In this case the deformity was of such character as to lead one to indulge in thoughts of the fashionable "fad"—the now popular operation, symphyseotomy. But even with this operation could our results have been better?

The papers were discussed by following members:

DR. L. E. NEALE: The instrument which bears my name is not original with me, but is a combination of the good points of many others. The blades are from Wallace's instrument and were selected because of their peculiar basket shaped curve which enables them to hold fast. The shanks are wide in order to conceal the traction rods, the attachment of which is very simple and rapid. The compression screw is placed at the end of the handle, out of the way and can be easily removed. It only has one ball joint but rotates completely. I agree with Dr. Williams, however, that it is best not to allow the instrument to remain on in rotated position. The principle of axis traction has come to stay, and we should have for general use an instrument admitting of axis traction application if desired. The four cardinal points obtained by axis traction, as laid down by Tarnier, should be at hand in one instrument.

Cases which originally present occipito-posterior positions will usually rotate anteriorly if let alone. Only about four per cent. of such cases remain posterior, hence they do not by their position alone indicate interference. In many of these cases the uterus may have clamped over the back of the child in such a way as to hold it no matter how the head moves. In a large majority of the four per cent. of cases, retention is due to lack of flexion. Now when we produce flexion, we have made the head smaller, so I differ with Dr. Williams's conclusions about a large head. We can try to get rotation by raising the brow or by use of vectis, etc., before resorting to forceps. Some cases will fail to rotate, no matter what method or what forceps you use, and will have to be removed with occiput remaining posterior.

DR. J. EDWIN MICHAEL: The present forceps are the outgrowth of a principle, the value of which has long been recognized. The introduction of Tarnier's forceps was a great step in obstetrics. By them you can make traction and not interfere with rotation, and you can apply your force in a direction in which it is needed. Your traction is in a line corresponding with the axis of the superior strait and in the direction which the head must follow. While the forceps do not facilitate rotation they do not hinder it. I believe that in most cases where rotation occurs with the instruments it would have occurred anyway, and we did not produce it. I have tried all the devices in those cases which persist in posterior position and yet they failed to rotate. When I deliver without rotation I always expect, and usually get, lacerations of perineum.

DR. CRAIGHILL: Dr. Michael has called to mind a case which illustrates well the the advantages of axis traction forceps. The patient had a contracted pelvis and the position was occipito-posterior. Both Hodges and Simpson's forceps were tried by Dr. Mitchell and myself, but both failed. We tried nearly all devices and were considering craniotomy when we concluded to try first Tarnier's forceps. Dr. Michael was sent for, supplied the forceps and delivered the child promptly and easily with one hand.

DR. W. S. GARDNER: The consideration of the mechanism of axis traction forceps includes the following points:

1. The pelvic curve of the instrument should be made to correspond as nearly as possible with the curve of the unyielding portion of the pelvic canal.

2. The cephalic curve should be so constructed as to give a firm grasp of the head of the child without too much compression.

3. The traction rod and handle should be so placed that traction will be made as nearly as possible in the direction of the pelvic canal.

4. The instrument should be made of material that can be sterilized by heat without injury.

Dr. Gardner then proceeded to explain the difficulty of determining the axis of the inferior strait and the fallacy of some of the usual statements made when considering the relation of the head to the pelvic outlet, and concluded that in so far as pelvic curves are concerned we can never do more than approximate the truth. As we cannot have a new instrument for each patient, the best we can do is to use that instrument which is most nearly correct for the largest number.

DR. W. E. MOSELEY: I wish to say a few words in defence of an old friend. It has been said here that the Hodge or Simpson forceps make an irreducible occipito-posterior position. I do not think that this is correct, for I have seen rotation occur with Hodges forceps on.

DR. P. C. WILLIAMS: Obstetrical mathematics would be very good if we could make the parts we are dealing with. In the majority of occipito-posterior cases rotation forward will occur but they create much suffering for the patient and I believe it is our duty to save as much pain as we can without danger to mother or child. So when I diagnose O. P. position, I think it best to terminate it as soon as I can safely. I am sure forceps do aid directly and forcibly in producing rotation.

H. O. REIK, M. D., Secretary.

525 *N. Howard St.*

